# “I’m just a long history of people rejecting referrals” experiences of young people who fell through the gap between child and adult mental health services

**DOI:** 10.1007/s00787-020-01526-3

**Published:** 2020-04-09

**Authors:** Rebecca Appleton, Farah Elahi, Helena Tuomainen, Alastair Canaway, Swaran P. Singh

**Affiliations:** Warwick Medical School, University of Warwick, Coventry, CV4 7AL UK

**Keywords:** Transition, CAMHS, AMHS, Narrative research, Qualitative research

## Abstract

The paediatric-adult split in mental health care necessitates young people to make a transition between services when they reach the upper end of child and adolescent mental health services (CAMHS). However, we know that this transition is often poor, and not all young people who require ongoing support are able to continue care in adult mental health services (AMHS). These young people are said to have fallen through the gap between services. This research aimed to explore the reasons why young people fall through the gap between CAMHS and AMHS, and what effect this has had on them and their families. Narrative interviews were conducted with 15 young people and 15 parents, representing 19 unique transition stories. Themes were identified collaboratively using thematic analysis. Reasons for falling through the gap were grouped into systemic problems and problems with the quality of care received. Effects of falling through the gap were grouped into separate themes for young people (feeling abandoned; struggling to manage without continued care; problems with medication) and parents (emotional impact of care ending; parents taking an active role in the young person’s care). To our knowledge, this is the first qualitative study that has focused only on the experiences of young people who have fallen through the gap between services. This research adds novel findings to existing literature regarding barriers to transition and the effects of discontinuity of care.

## Introduction

The period of late adolescence and young adulthood is often a time of several concurrent life transitions. For young people with mental illness, one of these transitions is moving from a child and adolescent mental health services (CAMHS), with an upper age boundary of 16–18 years, to adult mental health services (AMHS). As opposed to a simple transfer of care to a new service, the transition should be a “purposeful, planned movement” (1) encompassing the individual needs of each young person. This transition can be far from straightforward for young people and their parents or carers, who must navigate a complex system of care provision and negotiate the change from a holistic, family-orientated focus to one with a more individualistic, biomedical model of care (2).

It has been well-documented that transition from CAMHS to AMHS rarely goes smoothly, with young people experiencing distress, uncertainty, and discontinuity as they cross the service transition boundary (3–5). This is often due to young people experiencing disruption to their care, sometimes having several moves between services and/or clinicians during this time (6, 7) and struggling to develop new therapeutic relationships (8). The transition from CAMHS can also be a difficult time for their parents, especially if they are excluded from decisions around their child’s care once the young person is legally an adult (9). This can occur even if the young person is still reliant on their parents for support (10).

Whilst transition to AMHS can be difficult, some young people are unable to access specialist adult care, as they do not meet the eligibility criteria despite having an ongoing clinical need (3, 11). Sometimes, these young people are able to access third sector mental health support or wellbeing services such as IAPT (in this paper these will be referred to as Adult Wellbeing Services (AWBS)) (12). However, the availability of such services is not universal, resulting in a ‘postcode lottery’ when it comes to accessing appropriate mental health care (12). Young people who are not referred or accepted by an appropriate service after reaching the CAMHS/AMHS transition boundary are said to have ‘fallen through the gap’ between services. Currently, we know little about what happens to these young people after they leave CAMHS.

Previous qualitative studies exploring transition have focused on young people about to leave CAMHS or those who have transitioned to AMHS (e.g. 4, 13–15). There are practical reasons for this: recruitment usually takes place through a mental health service, which therefore biases research towards those receiving mental health care. The current qualitative study is linked to the MILESTONE Study (16); a longitudinal cohort study with a nested cluster randomised controlled trial, which followed young people who had reached the transition boundary of their CAMHS. This provided a unique opportunity to recruit young people who did not transition to AMHS. To our knowledge, this is the first qualitative study to focus on the experiences of young people who fell through the gap between mental health services. The aims were:

1. To investigate why young people with certain diagnoses fell through the gap between CAMHS and AMHS.
2. To explore the effect that falling through the gap has on the mental health and functioning of young people and their families.

## Method

This study is linked to a wider European project exploring young people’s transition from CAMHS to AMHS, details of which are reported elsewhere (16) and involved only the UK participants. Ethical approval was received from West Midlands—Black Country REC prior to data collection (REC Reference: 18/WM/0337). Informed written consent was obtained from all participants prior to interviews taking place. This study is reported following the Standards for Reporting Qualitative Research (17).

### Recruitment

Young people were recruited from an existing pool of participants from the wider study. It was decided to limit recruitment to young people who had been identified in previous research (carried out in the UK) as being most likely to fall through the gap between services: those with a neurodevelopmental, anxiety disorder, or depression (3). Those with emerging personality disorder were included due to previous contradictory findings (3, 18).

Young people were eligible to take part if they had one of the above diagnoses and had fallen through the gap between CAMHS and AMHS. Young people were said to have fallen through the gap if they were not referred to AMHS or AWBS after reaching the transition boundary despite having an ongoing clinical need, or if they were referred to an adult service only to be discharged by the next data collection point (6–9 months later) despite having an ongoing clinical need. The clinical need was indicated by a score of 2 or above on HoNOSCA questions (19) relating to psychological impairment.

If a young person was eligible to take part then their parent was also invited, providing they had consented to take part in the wider study. Purposeful stratified sampling (20) was chosen to ensure the sample contained a diverse group of young people and parents. Eligible participants were posted a study information pack and invitation letter, which was followed up by phone or text after two weeks if there had been no response.

### Data collection

**Fig. 1:**
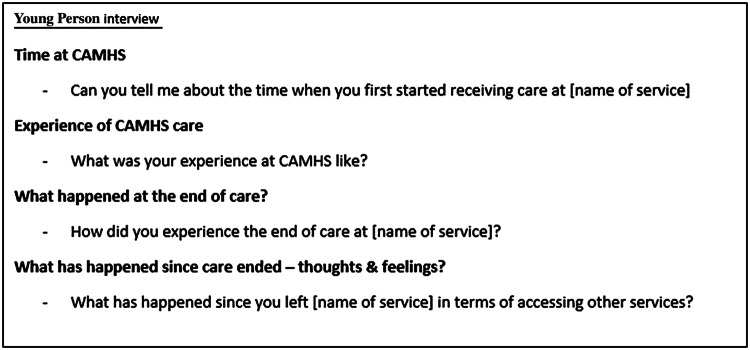
Topics and example questions used in narrative interviews with young people

Interviews were conducted by RA, a female researcher with previous experience and training in qualitative interviewing. A narrative interview technique was used to encourage participants to tell their stories (example questions shown in Fig. 1), followed by a period of purposeful questioning (21). The narrative method was chosen as hearing someone’s story is thought to be an effective way of finding out their lived experiences (22). Participants had the choice to be interviewed over the phone or in person, including the interview location, to minimise any discomfort or distress. All interviews were audio-recorded and anonymised during transcription. A sample size of 12–15 young people and 12–15 parents were chosen based on the principles of maximum variance sampling (23) and pragmatism due to the allocated time frame (this qualitative study was part of a broader research project linked to RA’s PhD thesis). RA kept a reflexive diary throughout data collection and analysis to help minimise the impact of any potential researcher biases such as previous research experience.

### Analysis

Interviews were analysed by two researchers (RA and FE) using thematic analysis as specified by Braun and Clark (24). Transcripts were read several times to ensure familiarity with the data and imported into NVivo v12 (25) for coding. The first round of coding consisted of a combination of descriptive, in vivo, and simultaneous coding (26). Themes were generated and refined iteratively through deliberation with the research team. Due to time constraints, participant checking was not used to validate the results (although participants were asked whether they would like to receive a summary of the results following data analysis).

### Findings

In total, 42 young people and 31 parents were invited to take part. Of these, 11 young people and five parents declined to participate, citing reasons such as a lack of time or the young person being too unwell. Fifteen young people and 15 parents took part, representing 19 unique individuals’ transition stories. In 11 cases, both the young person and the parent were involved. Four young people took part in the study without their parent, and four parents took part in the study without their son or daughter. Full demographic details for the young people linked with each transition story are presented in more detail in Table 1. Demographic details for parents were not recorded. In total, 25 interviews were conducted, five as joint interviews, and 20 as separate interviews with young people and their parents. One joint interview and six individual interviews were conducted by telephone, with the remaining participants interviewed in person. Face to face interviews took place either in the participant’s home or an alternative preferred location (e.g. library). In some instances, the parent was present for part or all of the young person’s interview, if this made the young person feel more comfortable. Individual interviews ranged from 14 min to 1 h 21 min (average = 36 min), whilst joint interviews ranged from 40 min to 1 h 22 min (average = 56 min).

**Table 1:** Demographic details of the young people linked with each transition story (*n* = 19)

Age in years, mean	19.42
Gender *n*, (%)	
Female	10 (53)
Male	9 (47)
Ethnicity *n*, (%)	
White British	17 (90)
British Asian	1 (5)
Mixed	1 (5)
Diagnosis *n*, (%)	
Mood and anxiety disorders	8 (42)
Comorbid autism and mood/anxiety disorder	5 (26)
Autism	2 (11)
ADHD	1 (5)
Other	3 (16)
Time since transition	
1–2 years	16 (84)
3–4 years	3 (16)
Current employment status	
University student	9 (47)
College/6th form student	3 (16)
Full time employment	3 (16)
Not in education, employment, or training	4 (21)
Current living situation	
Family home	10 (53)
University accommodation and family home	7 (37)
Moved out of family home	2 (11)

### Reasons for falling through the gap

Following thematic analysis, two main themes were identified that addressed the reasons for falling through the care gap (Aim 1): systemic barriers to continuity of care and problems with the quality of care received. These will each be described in turn, illustrated with examples from the transcripts.

### Systemic barriers to continuity of care

Systemic barriers to continuity of care captures anything related to the structure or culture of mental health services which makes it difficult for the young person to access appropriate care difficult after crossing the CAMHS transition boundary. There were three sub-themes identified: not being ill enough for AMHS; inadequate service provision after CAMHS; and a lack of joined-up care between services.

### Not being ‘ill enough’ for AMHS

The most commonly identified barrier to the continuity of care was young people being judged as not severely ill enough to access ongoing care. In some cases, their CAMHS clinician decided not to refer them to adult services, whilst in others, young people’s referrals to AMHS or AWBS were rejected following assessment. Young people were commonly told that they were ‘not ill enough’ to meet the threshold criteria to receive care at AMHS unless they were in crisis at the time of referral.

“…he hasn’t been able to access adult services, because what we’ve been told is that unless he attempts suicide etc., or in hospital, then he won’t be able to access the services.” [Parent (P)8 (Young Person (YP)7, Autism)].

It was also not enough for young people to have been at a crisis point in the months leading up to their transition. Two young people were not referred to AMHS despite attempting suicide in the months before their CAMHS care ended.

“And a couple of months before my care ended, I took a big overdose of my medication… they still said I wasn’t able to go to adult mental health.” [YP13, Comorbid autism & mood/anxiety disorder].

A common method of assessing illness severity and eligibility for a service was via a telephone assessment. However, these could be difficult for some young people, and resulted in further stress and anxiety:

“The thing is, it’s difficult… because I’ve got social anxiety and picking up that phone can be climbing a mountain sometimes, and certain days you just feel like, torture, talking to a stranger over the phone…” [YP9, Mood/anxiety disorder].

Telephone assessments were often described as short and impersonal, which raises questions about the suitability of this as a method for accurately assessing clinical needs.

“I think it was a 5 min phone call, I mean how can you assess someone’s mental health requirements with a 5 min phone call?” [P11 (YP9, Mood/anxiety disorder)].

There were also ethical issues regarding successfully managing the young person’s expectations and emotions during the assessment in a non-face-to-face setting:

“at the time I was really upset, I was really angry, I was really emotional, and they were just like ‘Well it’s the waiting list, we can’t do anything, here are numbers that you can call if you do feel low’.” [YP4, Mood/anxiety disorder].

Another common reason why young people were unable to access continued care was that care was often withdrawn during times of stability when they did not meet the treatment threshold.

### Inadequate service provision after CAMHS

Young people also reported struggling to find appropriate care which matched their level of need after leaving CAMHS. Some were offered a lower intensity of support, such as online or group therapy. However, all of the young people who were offered these types of therapy were reluctant to engage with them, believing these formats of treatment were not suitable and would result in further anxiety.

“it’s obvious that adult mental health don’t want me, and no matter how much you tell me to sign up to an internet, or talk anonymously to someone, I’m not going to do that, that’s not, me.” [YP6, Comorbid autism & mood/anxiety disorder].

In some cases, young people could not access any further care after leaving CAMHS, despite repeated attempts to reach out to AMHS or AWBS. For some young people, this lack of help was because there was no service suited to their level of need. This means they were stuck between being ‘too ill’ for AWBS, but ‘not ill enough’ for AMHS:

“Then it’s like adult mental health… they kept saying ‘You don’t meet that criteria’, … I tried to go to [AWBS]… but they wouldn’t take me on because I was fresh out of CAMHS and I was too big of a risk.” [YP13, Comorbid autism & mood/anxiety disorder].

Without mental health care, participants reported being signposted to other organisations if they needed help. Two parents were told to contact the criminal justice system if they required urgent help for their sons:

“So as I say, there’s no help there really for him, it’s just “Phone the police”, but that’s your son.” [PC8 (YP7, Autism)]

Young people and their parents were also told to go to A&E departments if they were in crisis:

“And then they just said that if you think that [Name] may harm herself, in terms of seriously harming herself, or somebody else, then to take her to the hospital. But, you know, have you ever tried to take someone to the hospital who’s suicidal? They just don’t go.” [P1 (YP3, Mood/anxiety disorder)].

In the absence of available NHS services, some parents paid for their child to receive help from a private counsellor. However due to the high cost of private appointments this was not accessible to everyone.

Nine of the young people who took part in the study were at University. These young people had the chance to access further care from University support services if they could not receive treatment on the NHS. However, the quality of care provided by universities varied considerably, young people describing mental health teams as *“very good”* or *“terrible”.*

### Lack of joined-up care between services

Participants also attributed their experiences to a lack of joined-up care between CAMHS and AMHS. This resulted in young people experiencing multiple transitions and contacts with different services after leaving CAMHS. Rather than being directly referred to AMHS, some young people were discharged back to their GP, for them to make the referral.

“At the end of care… we were told that we'd go onto adult mental health services. And we'd have to go back to our doctor… the doctor didn't really have a clue what we were talking about…” [P3 (YP4, Mood/anxiety disorder)].

This lack of direct referrals also led to some young people spending a significant amount of time waiting to access care, without being offered any alternative support during that time.

“…she just said to me that it would be a 6 month waiting list for group therapy and to see a psychotherapist.” [P3 (YP4, Mood/anxiety disorder)]

Some AWBS required a young person to self-refer to them, as opposed to being directly referred from another service. This was seen as a barrier for most young people who seemed reluctant to contact a new service themselves, despite having a need for continuity of care.

“and it was self-referral… So, getting round to that, it took a while after I turned 18.” [YP9, Mood/anxiety disorder]

In young people with more complex difficulties, mental health services were perceived as reluctant to take responsibility for that young person’s care, resulting in them being referred to several services without actually being able to access support.

“the doctors should have referred then him over to, for instance counselling, CBT, anger management, but he couldn’t do that because of the alcohol problem, and just said ‘Come back when you’re not drinking’.” [P8 (YP7, Autism)].

### Problems with the quality of care received

This theme captures any problems during a young person’s care at a mental health service which resulted in them falling through the gap. It is divided into three sub-themes: not receiving appropriate care whilst in mental health service; not prepared for CAMHS care to end, and put off accessing further care.

### Not receiving appropriate care whilst in a mental health service

Several participants reported not feeling as though they received appropriate treatment whilst at CAMHS. The lack of appropriate care may have contributed to young people falling through the transition gap.

“if they’d understood more about the condition I had more, that would have been a lot more helpful, earlier on, if they’d done that with me, rather than me having to go through 6 years” [YP10, Comorbid autism & mood/anxiety disorder].

In some cases this was linked with a reluctance of CAMHS clinicians to give a clinical diagnosis, which meant the young person was not able to receive the recommended treatment for their illness.

“because it [the diagnosis] took so many years, when they finally were like ‘Oh you need CBT therapy’, it was 5 years too late.” [YP2, Mood/anxiety disorder].

Some CAMHS were also criticised for relying too heavily on medication, as opposed to talking therapies:

“Especially, like my earlier team… they just kind of put you on medication and think ‘that’s that, you’re fine now’. They did do, therapy, like talking sessions but not many” [YP3, Mood/anxiety disorder].

This meant that some young people felt as though CAMHS never helped them to get to the core of their symptoms. Therefore, despite having sometimes lengthy care at CAMHS they were still struggling with their mental health when they reached the upper age limit of the service.

“And when we left CAMHS, again they said ‘Oh I’m sure it will be alright now’ – how can it be alright if they never got to the root of the problem?” [P1 (YP3, Mood/anxiety disorder)].

In the majority of cases, young people who were able to access ongoing care at AMHS or AWBS also experienced a poor standard of care that did not meet their needs.

“I think he’s now with I-A-P–T service, just looking at the card he’s got pinned up. He’s got one more session tomorrow, so basically they’ve done nothing either.” [P6 (YP not interviewed, Comorbid autism & mood/anxiety disorder)].

A particular problem identified was the infrequent nature of appointments at AMHS or AWBS, in some cases with young people waiting months between sessions, which meant they did not find them beneficial.

### Not prepared for CAMHS care to end

Some young people did not feel prepared for CAMHS to end and did not know where else they could access care or go to for help. Consequently, they struggled to manage on their own. In most cases, young people did not feel adequately prepared due to a sudden cut off of care at CAMHS and withdrawal of all support.

“I just think, I mean there’s no reduction, it’s like with medication, you don’t just stop medication, you reduce it. Saying that you’re 18 now, sorry, bye.” [P6 (YP not interviewed, Comorbid autism & mood/anxiety disorder)].

This poor planning meant that young people felt rushed and under pressure to make a decision about their future care, in some cases in the absence of receiving appropriate information about where they could go next. Young people commonly reported only being given information leaflets, with no opportunity to discuss options with their clinician. Some young people also reported not being involved in the decision making process regarding their end of care at CAMHS, meaning they were not informed about future care options.

In contrast, a few participants were very well prepared for care to end, which helped them and their parents manage their illness after leaving CAMHS.

“He gave us lots of advice of places to turn to didn’t he? He did advise us it was going to be a rocky road probably, it wouldn’t all be plain sailing.” [P15 (YP15, Other)].

### Put off accessing further care

Having bad experiences of care at CAMHS or having referrals to other services rejected led to some young people being put off from accessing further care, despite needing further support.

“because she had such a bad experience, she didn’t want to see anyone” [P1 (YP3, Mood/anxiety disorder)]

Young people who did try to access services after CAMHS found it difficult to have to repeat their story each time. Several participants emphasised the importance of having a trusting relationship with their clinician, which could take a long time to form and made them reluctant to have to ‘start again’ with someone new:

“I have to get to know someone before I’m going to talk to them first, and I want to know as much about them, the individual, as they want to know about me. To me, they’re a stranger, whether they’re a professional or not.” [YP7, Autism].

Several young people felt that there was no point engaging with care after CAMHS if their referrals were always going to be rejected or if they found the service to be too impersonal and different to CAMHS.

“And some of the doctors who I dealt with at [AMHS], they were just kind of so half hearted, I always felt as though I was being a bother to them just by showing up, and that just made me feel so bad.” [YP1, Mood/anxiety disorder].

There were also cases of young people being put off engaging with further care because they wanted to be in control and have care ending on their terms.

“Because if it comes to the part where you tell me I’ve got to let go I don’t think I will be able to, so I’d like to do that on my terms… she was like ‘I can offer you two or three more sessions’ and I was like ‘I don’t think I want to go that far and then for you to tell me that I can’t come back’.” [YP4, Mood/anxiety disorder].

In one case, this was also linked with a desire not to be stigmatised:

“And I think once he became older, his concern was the fact that being a male, being a black male, that statistic basically, he didn’t want to fall into that statistic, and so he just wouldn’t engage.” [P9 (YP not interviewed, Other)].

### Effects of falling through the gap

The findings linked to the effects of falling through the gap (Aim 2) are divided into separate themes for young people and parents.

### Effects on young people

The effects on young people are categorised under: feeling abandoned, struggling to manage without continued care, and problems with medication.

### Feeling abandoned

Most young people reported feeling abandoned; that they had been let down by the system and no one cared about them. The language used by young people emphasised their feelings of abandonment, with phrases such as *“*lied to*”*, *“*pushed into the wilderness*”* and “shut the door on me*”* used to describe how they felt about what happened when their CAMHS care ended. This language also suggests that young people did not have a choice as to when their care would end and what happened next. As health services were perceived as uncaring, most young people reported feeling as though they were ‘on their own’, without any support.

“I suppose they’re meant to transfer you to other care things, but it was more like “you’ve got to do it yourself now”” [YP3, Mood/anxiety disorder]

In some cases, feeling abandoned by mental health services had a negative impact on their mental health, in particular when young people were told they were *“*not a priority*”* by clinicians. This led to some young people feeling as though they were not worthy of help and questioning if they would be able to get better.

For some young people, feelings of abandonment were intensified as CAMHS ended at a time when they needed help the most due to other, stressful life events. This led to increased anxiety and a loss of confidence in how they would cope with this stressful period alone.

“I was going through a court case at the time, it had only just started, and I needed someone really to talk to about that. But then, being… erm, I was like sent away from CAMHS, there was no one there.” [YP13, Comorbid autism & mood/anxiety disorder].

### Struggling to manage without continued care

The majority of young people struggled to manage on their own after leaving CAMHS. In the most severe cases, young people were viewed by their parents as missing out on normal life due to a lack of appropriate support:

“he’s not living the life that he should be living, I do feel he’s being let down. He never leaves the house now, he hasn’t left the house since April [10 months before the interview]. But he’s not the person he should be and he does need help…” [P8 (YP7, Autism)].

Others were taking time out of work, education or training due to struggles with their mental health.

In contrast, a few young people were able to manage their mental health well without the need for continued care. For these young people, having very supportive parents was critical to them being able to manage, for example, one young person required extra support from her parents when away at university:

“For people like [Name], there’s no transition, there’s nothing. She is very lucky, in the family she’s got, without blowing our trumpets, we would drop anything to be there.” [P5 (YP6, Comorbid autism & mood/anxiety disorder)].

The majority of young people coping without care were prepared well for CAMHS to end, which seemed to increase their confidence in their abilities to manage without professional support.

“because he gave us plenty of warning I had time to ask questions and sort of plan strategies for how we would cope, the practicalities of it.” [YP15, Other].

Despite being officially an adult, the majority of young people felt as though they would still need help from their parents when it came to organising care post-CAMHS. This suggests that although they are legally viewed as an adult, young people with mental illness continue to rely on their parents for support.

“obviously my mum did it all for me, it scared me the fact that I’d have to do it myself, because I’m not great with things like that.” [YP3, Mood/anxiety disorder].

### Problems with medication

Almost all young people who were taking medication at CAMHS encountered various problems in accessing, changing or stopping their medication after leaving the service. For some young people, falling through the gap meant they had no choice but to stop their medication once their existing prescription ran out.

“I’m not on medication now, and I haven’t been since I was 18, basically since I needed to be seeing a doctor to be able to be prescribed more meds, I didn’t have one at the time.” [YP1, Mood/anxiety disorder].

Although some young people were able to continue taking their medication, they were not given information about what to do if they wanted to change their dosage or stop the medication altogether.

“I’m not sure. I haven’t had a medication review for a… ages actually. So I probably could go to the Doctors down here, but then again, last time I went to discuss about my medication they said they couldn’t do it, so, I’m not too sure.” [YP13, Comorbid autism & mood/anxiety disorder].

Some young people who were coping well without any support apart from medication were also unsure whether they should still be taking it, and how long they should be taking it for.

### Effects on parents

The effects on parents of their child falling through the gap are divided into two themes: Emotional impact of CAMHS care ending and parents taking an active role in the young person’s care.

### Emotional impact of care ending

All parents interviewed spoke about the emotional impact that the end of their child’s care at CAMHS had on them. In particular, parents saw CAMHS as a ‘safety net’ and since leaving CAMHS they worried about what would happen in the future, especially if their child’s mental health deteriorated.

“I think it would be nice to have like, a sort of contact person, in that department that you could call and say ‘She’s relapsed, can we access somebody, a specialist immediately, rather than having to go back through the Doctor to be referred because obviously if you relapse you almost need immediate help, don’t you?” [P15 (YP15, Other)].

For some parents, these feelings were exacerbated as the end of CAMHS came as a shock. One parent, in particular, was surprised when CAMHS ended when his daughter turned 16, less than a year after she began receiving care:

“we’d just worked out the routine and what was going on, and it was pulled from under us, so it was, that was part of the shock really, that thing had gone, as it had only just got going really.” [P2 (YP not interviewed, Mood/anxiety disorder)].

Many parents also reported feelings of frustration due to the poor experiences of transitional care. In some cases, parents were also frustrated as they were left out of decisions about their child’s care after CAMHS. Parents reported feeling “so outside of it all” and excluded from information about their child’s health.

### Parents taking an active role in young person’s care

In the absence of professional support, parents reported taking an active role in their child’s care. At the most extreme level, this meant they took on the role of ‘Doctor’, helping to implement coping strategies for their child or helping to wean them off medication when they were unable to renew their prescription.

“I weaned myself off it, I mean with my mum’s help and supervision. So I started taking less, a lower dosage over time so that I could wean myself off it, because we discovered that I wouldn’t be able to get another prescription without my doctor seeing me, and my doctor wasn’t seeing me because the CAMHS people wouldn’t see me again.” [YP1, Mood/anxiety disorder].

In the less extreme cases, most parents reported helping their child to access care, for example by making GP appointments for them or finding out information regarding what care they could access at university. However, several parents reported difficulties in helping their child access support once their child had turned 18. Many reported having to fight to access the appropriate care for their child.

“I’m not sure how my mum managed to get me to [Service], but I’m pretty sure in the end she just annoyed them so badly by directly contacting them that they begrudgingly gave us an appointment.” [YP1, Mood/anxiety disorder].

In some cases this led to parents feeling as though they were being labelled as “overprotective” or “paranoid” by healthcare professionals, although this did not deter them from continuing to push for help.

“As parents we struggle. We don’t know whether we’re doing right, we don’t know whether we’re doing wrong. It’s difficult to explain to the GP, it’s difficult to explain to everybody. They think you’re just being awkward, overprotective.” [P4 (YP5, Mood/anxiety disorder].

Some parents also spent a significant amount of time providing emotional support for their child, going above and beyond a usual parent–child relationship. Most young people emphasised how important they found the support of their parents in the absence of professional support, showing the value of having a supportive family to help young people cope with their mental health.

“I’m really lucky that I’ve got really loving parents who look after me and make sure that I’m not in danger, but if I had any less of a support system I’d probably be dead by now because it took them so long to do anything.” [YP1, Mood/anxiety disorder].

## Discussion

This research is the first of our knowledge to explore the perceptions and experiences of young people who had fallen through the gap between CAMHS and AMHS, as well as the views of their parents, up to three years after leaving CAMHS allowing for long-term reflections of their experiences. Overall, the results suggest that there are still significant problems with the transition process, despite over a decade of transition research and the introduction of policy guidelines (27).

**Fig. 2:**
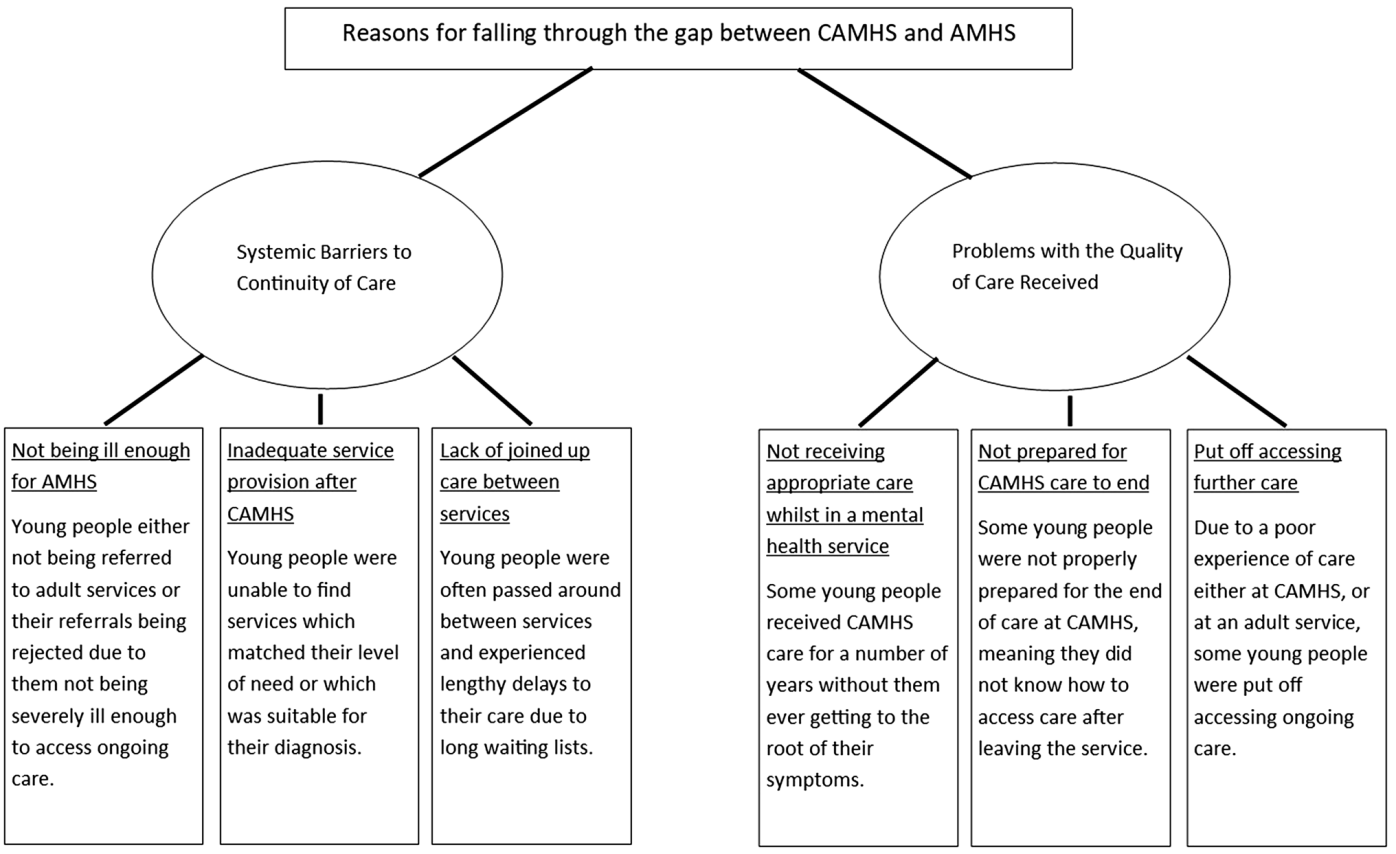
Diagram showing the themes and subthemes for young people and parents views as to why young people fell through the gap between services

Reasons for falling through the gap were split into systemic barriers to continuity of care and problems with the quality of care received (see Fig. 2). Young people’s gender, ethnicity, diagnosis, living situation or employment status did not appear to affect the reasons for falling through the gap. One of the most common reasons for young people falling through the gap was them not meeting the threshold for entry into AMHS, something which has been previously identified by other studies (3). An important finding of the current research was the identification of a group of young people who were not ill enough to access AMHS, but were too ill for other/non-specialist AWBS, meaning they were left without any professional support. This relates to findings from previous research regarding experiences of young people with ADHD, who struggled to access care which suited their needs after leaving CAMHS (28). Filling this gap in service provision should be a priority for future care initiatives.

Some of the barriers identified by young people to access AWBS were the need for self-referral and the frequent use of telephone assessments to assess eligibility. For some young people this caused further anxiety, and resulted in a reluctance to engage with care, despite requiring support. Several young people disliked the use of telephone assessments, feeling as though they found it difficult to express themselves over the telephone in a short space of time. For some participants, these assessments caused significant distress, which raises ethical issues around managing the emotions resulting from assessments outside of a clinic setting.

Another commonly identified (8, 28) barrier to ongoing care was a reluctance of young people to see a new clinician and build a new therapeutic relationship. Young people also described a reluctance to engage with a group or online therapy offered after CAMHS, describing them as too impersonal or unsuitable for their needs. These findings contradict previous systematic reviews that have found good levels of acceptability for group therapy for adults with anxiety (29) or online therapy for adults with anxiety or depression (30).

In the absence of specialist mental health support, some young people and parents reported being told to go to A&E if they needed urgent mental health care. Other parents were told to contact the criminal justice system if their child was in crisis. These findings support previous research that has attributed A&E and police involvement in mental health crisis care to the decline in AWBS (31, 32) and shows that the shortfall in mental health services can result in increased use of resources elsewhere. Future policy guidelines should focus on ensuring appropriate mental health crisis care is available to those who need urgent support.

Young people and parents attributed a poor standard of mental health care as one of the reasons why they had fallen through the gap between services. One novel finding from this study was that some young people were dissatisfied with the quality or frequency of care received at CAMHS, feeling as though CAMHS had not helped them improve their mental health, despite years of treatment. This resulted in them still requiring support after leaving the service. Other participants instead cited the poor standard of care from AMHS or AWBS as a reason for why they fell through the gap. There was an overall dissatisfaction with mental health care after CAMHS, with infrequent appointments and clinicians perceived as uncaring.

Those who were not referred to other services after CAMHS were discharged to their GP. Care under GPs was variable: some young people felt well supported, whilst others thought their GP did not have the appropriate expertise to manage their medication or make appropriate referrals to other services. This finding has implications for clinical practice, in particular regarding adequate training of GPs in managing common mental health conditions. Other recent research in the UK has identified a lack of GP training in managing young people’s emotional distress, suicidality, and ADHD (33–35).

**Fig. 3:**
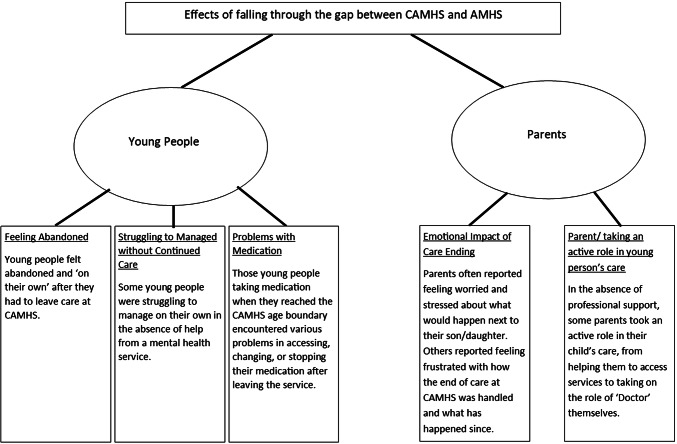
Diagram showing the themes for the effects of falling through the gap between services

Falling through the gap had overwhelmingly negative effects on young people and their parents (see Fig. 3). In the most severe cases, young people struggled to manage on their own, which resulted in them missing out on education, work, or friendships. Most young people reported feeling abandoned by services, something also reported in other qualitative studies exploring young people’s experiences of transition (5).

Young people also commonly mentioned problems with medication after leaving CAMHS. Previous studies that have explored medication use after crossing the transition boundary have focused on whether young people are still able to access medication after leaving CAMHS (36). A new finding from this research is that those who remained on medication also experienced problems after crossing the transition boundary, something which has not been found in other qualitative studies of young people. Several young people had not had their medication reviewed since leaving CAMHS, and were unsure about whether they were receiving the correct dosage or whether they should still be taking their medication. This finding mirrors the results of a qualitative study exploring the role of GPs in prescribing ADHD medication for young people after CAMHS, with some GPs unsure if they had the appropriate expertise to prescribe specialist medication without support from secondary care (37).

In the absence of professional support, parents reported taking on a significant role in their child’s care. This ranged from trying to identify appropriate services for their child, to taking on the role of ‘Doctor’ themselves to provide therapy or wean their child off their medication. This was despite some parents feeling as though services were labelling them as overprotective, which reflects a disconnect between the clinician and parent’s view of the severity of the young person’s mental illness. This finding also raises the questions of who is there to support these parents, and whether they have the correct knowledge to provide this level of support to their child. It also highlights the potential economic impact of a young person’s mental illness on those around them. Future health economic evaluations of mental health interventions should attempt to capture this spillover effect on carers and family members.

Parents of young people who had fallen through the gap often experienced worries about their child’s mental health and what would happen if they deteriorated and were not able to access services. Consistent with other literature (9), parents also reported feeling excluded from the transition process, which resulted in uncertainty about their child’s care and how they could best support them. The results of the current study suggest that parents of those who fell through the gap are stepping in at the point where they would normally be taking a step back: once young people transition to AMHS, it is unlikely that their parents are able to be involved in their care (4). As parents are providing significant support for their child after CAMHS, it suggests that these young people were not ready for care to end.

## Strengths and limitations

One of the main strengths of this study was that we were able to recruit young people who had fallen through the gap between one and three years after leaving CAMHS. This allowed us to explore the long-term impacts of falling through the gap on young people and their families. The data collected was extremely rich, and we were able to capture a variety of different transition experiences. There was also no observable difference in the depth or quality of interviews conducted face to face or over the telephone. This could be due to participants choosing the method of data collection: some may have felt more comfortable disclosing sensitive information over the telephone (38).

This study also contained some limitations. There is a potential for response bias from participants, as those with a particularly bad experience of transition may have been more willing to take part. The data collection method may have discouraged some from taking part, as some potential participants declined to participate as they were too unwell. Almost 50% of the participants were at university, meaning the sample may be biased towards those who are more highly educated. The majority of participants also still lived in the family home (either permanently or during university holidays), suggesting that this sample may have been biased against those without a strong family support network or those without a fixed address. The length of time since leaving CAMHS and the interview may have affected the accuracy of participant recall. However, as the research focused on how participants experienced the end of care at CAMHS, the overall accuracy of the data is not a significant cause for concern. There were also some discrepancies between the diagnoses obtained at the start of MILESTONE from either their CAMHS clinician or clinical records, and the diagnoses identified by the participants themselves. This could be due to diagnoses naturally changing over time, or a misunderstanding between the clinician and participant, and likely reflects the challenges of diagnosing adolescents and young adults. It was decided to report all participant-reported diagnoses, as these were likely to be the most up to date, although this may not reflect their formal clinical diagnoses. As many of these young people were no longer registered with a mental health service, we were unable to obtain up to date diagnostic information.

## Conclusions

Our findings confirm that young people fall through the mental health service gap due to a lack of available and appropriate care after leaving CAMHS, which can result in significant anxiety for young people and parents. Other reasons include a lack of appropriate care from mental health services, and young people being put off accessing further care due to anxiety about self-referrals and telephone assessments, or poor experiences whilst trying to access care. A small number of young people were struggling to cope on their own. Managing medication after leaving CAMHS poses a struggle for several young people. In the absence of professional support, many parents take on the responsibility for their child’s mental health. This study also identified a group of young people who fall between the eligibility thresholds for AMHS and AWBS. New models of care for transition-aged youth should focus on filling this gap in service provision.
